# Prevalence and predictors of suicide ideation among university and high-school students during India’s 2nd wave of the COVID-19 pandemic

**DOI:** 10.1371/journal.pone.0311403

**Published:** 2024-12-05

**Authors:** L. M. Frey, Devi Venugopal, Varsha S. Dev

**Affiliations:** 1 School of Social & Behavioral Sciences, Amrita Vishwa Vidyapeetham, Kollam District, Kerala, India; 2 Department of Psychiatry, Father Muller Medical College, Mangalore, India; 3 Department of Amrita Lead, Amrita Vishwa Vidyapeetham, Kollam District, Kerala, India; Mattu University, ETHIOPIA

## Abstract

Student suicide ideation increased globally during the SARS-CoV-2 pandemic. There is scarce literature addressing suicide ideation during global health crises. Therefore, this study investigates prevalence and predictors of suicide ideation during India’s COVID-19 Second Wave. We also examine the 3-Step Theory’s assertion that both pain and hopelessness are necessary to have suicidal thoughts. Sample recruitment was through an online anonymous questionnaire. Inclusion criteria included students aged 18 or older, living in India during the time of the study (*N* = 535). Data collection was through the online questionnaire. Three categories of variables were investigated as potential predictors of suicide ideation: Sociodemographic (e.g., age, gender, education, economic status), COVID-19-specific (e.g., online classes, fear of virus contagion, vaccination status) and Clinical (e.g., sad mood, fear, loneliness, hopelessness). Data analysis (using SPSS-26) included descriptive statistics for describing data characteristics, Spearman Rho Correlation (assess the strength and direction of association between variables), and Binary Logistic Regression to help identify predictors of suicide ideation. Ordinal variables were measured using Likert scales with some recoded into binomial variables for the Regression analysis. Clinical variables predicted suicidal ideation, including fear of failure (OR = 4.17, 95% CI:2.51–6.94; *p* < .001), sleep disturbance (OR = 3.04, CI:1.67–5.52; *p* < .001), loneliness (OR = 2.77, CI:1.21–6.32; *p* < .01), sadness (O = 2.89, CI:1.59–5.32), and loss of interest (OR = 2.60, CI:1.37–4.93). Suicidal thoughts were reported by 48.7% of the student-participants. The Three-Step Theory was partially supported, as students feeling psychological pain but not hopelessness still reported suicidal ideation. Anticipating future global health crises, policy-supported mental health mitigation strategies are critically needed for youth, designed to reduce suicidal ideation, enhance resiliency, and to foster mental skills. These should enable them to successfully manage unexpected life challenges.

## Introduction

The SARS-CoV-2 pandemic had a strong influence on suicidal behaviors in many countries. Suicide ideation typically precedes an act of suicide, which is among the most tragic causes of death among youth. A recognized global health crisis [1,2], suicide is the fourth leading cause of mortality among those aged 15 to 29 [3], marking this age group as a vulnerable population. The World Health Organization (WHO) reports that 800,000 to one million self-inflicted global deaths occur annually. Yet many researchers consider this to be an underestimate due to underreporting [4], misattributing suicides as unintentional injuries and “other causes” [5], concealing suicides due to family stigma [6–8], and because only 15% of WHO’s 194 member states verify vital data registration [4,8]. For example, one study reported that 87% of deaths labeled as “undetermined” had evidence of suicidal intent [9]. Underreporting self-inflicted deaths distorts information that would otherwise be used to inform policy, research, treatment interventions, and preventative actions.

Suicide ideation (SI) is a predictor of suicide attempts [10–13]. It has a crucial role in understanding the process of a person transitioning from suicidal thoughts to a plan, to action [10,11,14,15]. Research reports that 33% of those with SI will make an attempt [16,17]. For example, a 13-year study found that those with SI at baseline were more likely to have made attempts at the follow-up assessment [18]. This helpful knowledge emphasizes the importance of studying suicide ideation because historically, it has been difficult to identify consistent indicators that predict suicidal behaviors [19–22].

This study examines suicidal ideation among school and university-attending youth in India during the SARS-CoV-2 pandemic. This is an important undertaking for at least four reasons. First, there is a notable increase in SI onset between the ages of 18 and 35 [16], a sensitive age period that warrants further study. Yet, even young children experience suicide ideation as early as age 5 [23,24]. Second, during the first year of SI onset, the risk of transitioning to a plan or attempt is at its highest [16,23] underscoring the importance of recognizing, evaluating, and acting upon suicide ideation quickly. The third reason for studying youth SI is that the COVID-19 pandemic triggered numerous stressful situations around the world. These circumstances are important to understand as they led to a rise in suicidal behaviors, particularly among youth and marginalized communities. Fourth, there is a dearth of information regarding suicide behaviors that occur during large-scale, unprecedented disease outbreaks. For these reasons we assessed student suicide ideation during India’s COVID-19 2nd Wave. To create effective solutions in anticipation of future pandemics and other emergencies, it can be helpful to understand the factors that increased SI among youth during the pandemic.

From here, section 2 provides a brief overview of prevalence rates of suicide mortality in India, and the context of India’s second wave of the pandemic. Section 3 introduces the Three-Step Theory of suicide behaviors, and the objectives of the study. Section 4 outlines the methodology. Our findings are reported in Section 5, which is followed by the Discussion (Section 6).

### Suicide mortality in India

Suicide mortality is high in India compared to other countries. Between 1990 and 2016, suicides in India increased by 40% [25,26]. Student suicide rates steadily increased during the last three decades. Between 1995 and 1999 the reported total number was 27,359. This figure spiked to 48,537 between 2015 and 2019. In 2019 the one-year student suicide rate, 10,335, was higher than any other in the prior 25 years [27]. Despite these somber figures, experts question the accuracy of India’s suicide prevalence reported by the National Crime Record Bureau (NCRB) [27]. For example, in 2016 there were 131,008 NCRB-reported deaths by suicide [27] compared to 230,314 reported by a Global Burden of Disease study in India [26], a difference of 54.97%. Yet the suicide reports were even higher during the pandemic. We now address the COVID-19 pandemic and its influence on suicide ideation.

### The contextual situation during India’s 2nd wave

Internationally, government responses to curb COVID-19 viral transmission included multiple constraints and directives: Mass lockdowns, restrictions of large gatherings and mobility, and public safety health awareness to prevent viral contraction (handwashing, mask-wearing) [28]. India’s response to the pandemic was one of the world’s most restrictive as measured by the Oxford Coronavirus Government Response Tracker “Stringency Index” [29]. The index is a composite of nine restrictions: public gatherings; international travel; closures of: jobs, schools, public transport; canceled public events; restrictions on internal movement and public information campaigns; and stay-at-home orders [29].

**Fig 1:**
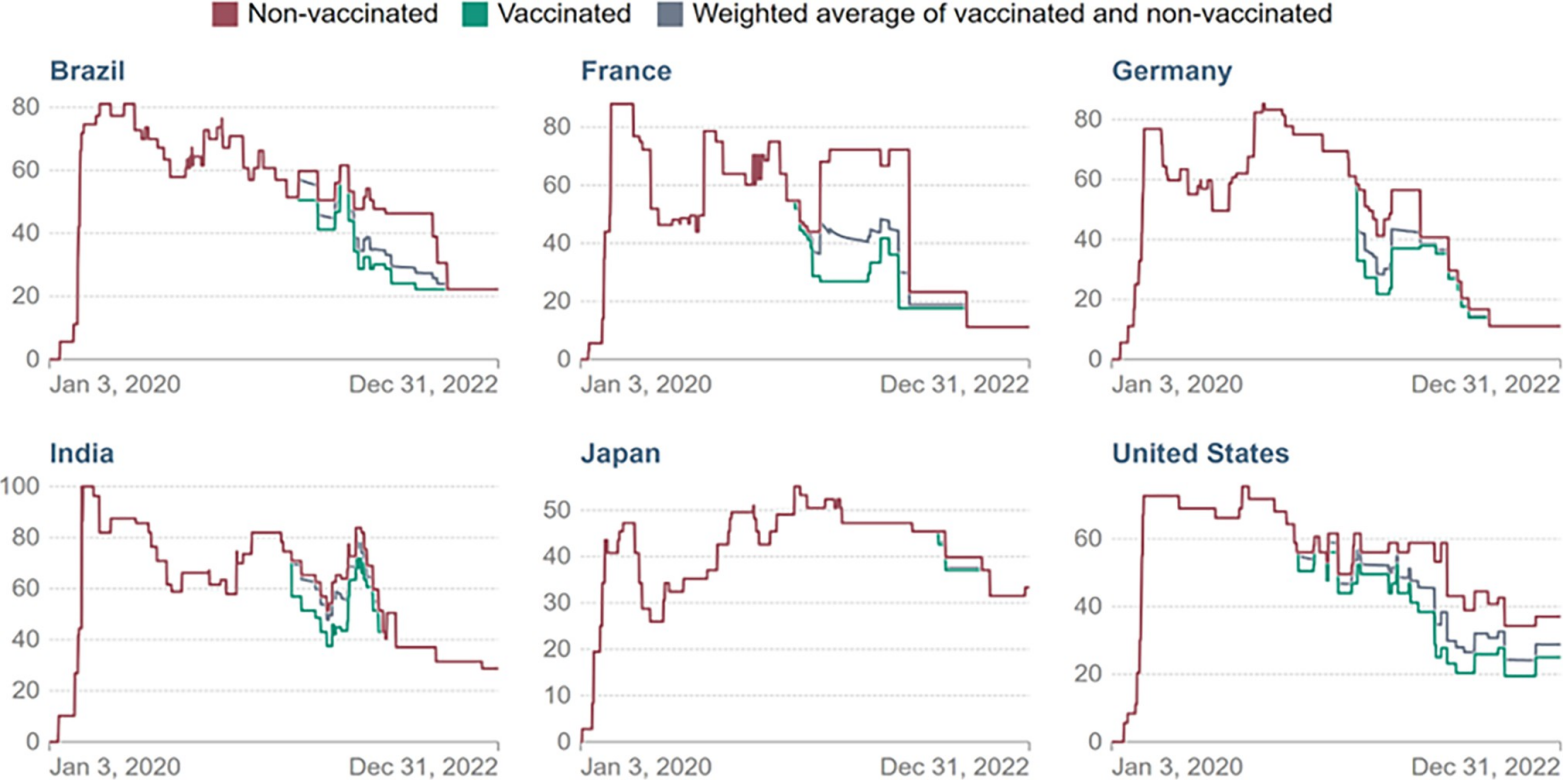
COVID-19 stringency index of top 6 countries with highest mortality. (Fig 1 courtesy of Hale et al., 2021 [29]: Oxford COVID-19 Government Response Tracker).

A very high stringency index suggests greater restrictions upon a populace as noted below. Fig 1 indicates that among the top six countries with the most reported COVID-19 cases in 2022, only India’s stringency index reached the 100% mark (Y-axis) [30]. Brazil, France, and Germany hovered between a score of 60 to 80, whereas the US held to a level of 65, then reduced steadily through the year to a low index between 5 and 30 (blue line). Japan, by contrast, never reached the score of 60, and mostly hovered around an index of 40 to 50.

Did a country’s stringency index level influence percentages of COVID-19 cases? The US has one-fourth the population of India but reported 60 million more COVID-19 cases. At first glance this suggests that the more stringent lockdowns in India mitigated the spread of COVID-19. However, alternate explanations were suggested by scientists and journalists who provided evidence of the country underestimating both COVID-19 cases and mortality rates by up to 30 times higher than what was reported [31,32]. Overall, stringency measures attempted to reduce a rapidly advancing lethal virus. But it was at the economic expense of citizens who lost income due to the closure of non-essential businesses, a bitter choice with risks and repercussions both ways [33]. Education was also heavily impacted, as we now demonstrate.

#### Education constraints

Education expands youth employment opportunities which can lead to economic security. School attendance provides opportunities for youth connections, socialization, and creative learning endeavors with teachers and friends. Overall, approximately 1.6 billion students from 190 countries [34] were affected by the pandemic. In India, the mutating, persistent COVID-19 virus and lockdowns forced nearly 300 million students to rely on online platforms to continue their education [35]. This was a humongous task requiring massive efforts to rapidly integrate educational technology into institutions [36].

Millions of people were deprived of their usual income due to lockdowns, leaving innumerable families without the resources to provide computers or smartphones to their children for online classes. Moreover, only 45.0% of India’s population had internet access as of January 2021 [37]. These factors magnified the educational disparities between the economically stable and those more marginalized, particularly in rural and poor regions. Consequently, millions of youth had no digital access, and reports of student suicides haunted the nation [38,39]. Reasons for the suicides included a fear of losing access to education, inability to take exams, fear of exam failure, and poor networks that interrupted class participation and learning [40].

Additionally, for many children problems also erupted on the home front. Families are theoretically safe harbors for their members. But during the pandemic many women and children were reportedly subjected to violence and maltreatment [41,42], which can substantially affect a child’s capacity to learn [43].

#### India’s 2nd wave

Even as all of the above-mentioned educational problems were happening, in early 2021 the country was more confident, having successfully contained the virus during the initial year of the pandemic (2020). This was partly due to good practices and outcomes achieved in the state of Kerala [44] which other states then adopted.

However, as other countries had also done, in early 2021 the lockdowns were prematurely relaxed and the government opened the doors to unrestricted public events: marriages, huge election rallies, public festivals, and other social functions [45]. This led to the tragic second wave of the pandemic. The sudden removal of social restrictions led to massive surges of COVID-19 infections, up to 300,000 daily. This unanticipated astronomical number of cases led to national acute shortages of hospital beds, oxygen, ventilators, other essential equipment, and antiviral drugs. In spite of a multitude of courageous, dedicated medical staff and personnel, the shortage of beds led to many ill patients dying outside of hospitals while waiting for treatment [46]. Independent researchers such as the Center for Global Development reported millions of deaths beyond those officially disclosed [47]. There were insufficient crematorium resources to keep pace with the innumerable deaths, necessitating vast numbers of mass outdoor cremation sites [48]. Grief and fear gripped the nation. Mental distress soared.

The accumulation of the above-mentioned factors contributed to increases in suicides and declines in mental wellbeing. Mental health problems increased by 35% during the protracted pandemic [49]. In 2020 alone a 10% increase in self-inflicted deaths contributed to an average of 400 suicides daily (146,000 annually), with student suicides being among the highest [50]. Alarmingly, this figure again rose by 7.1% the following year to 164,033 [51]. Even prior to COVID-19 the United Nations pressed all nations to reduce suicide mortality by 33% by the year 2030 [52,53]. This is an ambitious yet crucial goal to mobilize further understanding of the underlying causes of this heartbreaking problem [8]. Experts warned that if India’s recent alarming suicide surge continued, the country would have a “zero probability” of meeting the United Nations goal [26]. Given these multiple national challenges during the pandemic, and the scarcity of data on suicide behaviors during large-scale, protracted disease outbreaks, we assessed student suicide ideation during India’s COVID-19 2nd Wave. From here we introduce the 3-Step Theory (3ST) of suicide behaviors, the objectives of our study, and the study methods.

### Theory of suicidal ideation

Although said to be preventable, suicide remains shrouded with complexities after a century of study. Recent theories propose meaningful steps between the transition from suicide thoughts to suicidal actions. One of these is the Three-Step Theory (3ST) [16] which has three progressive steps. These are: 1) suicide ideation, 2) presence or absence of connectedness, and 3) dispositional capacity to commit lethal self-harm. Step 1, suicide ideation (SI), is our focus. The 3ST claims that SI is influenced by the paired experience of pain (psychological and/or physical) and hopelessness. The authors, Klonsky and May [17], suggest that pain alone is not likely to lead to suicide ideation. But if the person also experiences hopelessness, then the risk of experiencing SI increases. Klonsky and May [17] suggest that heightened SI can be mitigated by connectedness (step 2) which they define as an active interest, hobby, or supportive people. Yet in the absence of connectedness suicide ideation is at risk of escalating. This, in turn, elevates the risk of a person considering and/or making a suicide plan and attempt. A suicide attempt is particularly more likely by those whose temperament is unintimidated by the act of committing suicide (step 3).

Our study aims to determine predictors of SI during a tumultuous time in India, and to examine the first step of the 3ST: Whether psychological pain and hopelessness coexist and determine reported SI among participants, or whether SI can occur without one or both of these two variables being present. Therefore, in this study we examine potential predictors of suicide ideation, and we also test the 3ST theory with regard to paired pain and hopelessness as a predictor of SI.

### Study objectives

Given the highly unusual global circumstances of the COVID-19 pandemic and related national and international restrictions, we sought to determine possible pandemic-related variables and research-supported variables that could be predictive indicators for youth SI. We also tested the 3ST theory regarding paired pain and hopelessness.

#### Objective 1

What are the sociodemographic, COVID-19-related, and research-supported ‘Clinical’ predictors of SI among this sample of students? Is there a model related to these variables that contributes to our understanding of SI among students during the pandemic?

We hypothesized that specific COVID-19 related variables and Clinical variables would predict suicide ideation (e.g., economic vulnerability, viral fear, loneliness). We also hypothesized that loneliness would be among the strongest predictors given the social isolation induced by necessary enforced lockdowns.

#### Objective 2

Do the identified predictors support the first step of the 3ST of suicidal behaviors in terms of combined hopelessness and pain? Drawing from 3ST theory of suicide, we questioned whether combined pain and hopelessness would co-exist among our sample. We also questioned whether these were necessary to experience suicidal thoughts, or whether SI can occur without these two variables being present.

## Methods

### Ethics

The procedures involved in this study comply with, and are consistent with, ethical standards of the relevant national and institutional committees on human experimentation and with the Helsinki Declaration of 1975, as revised in 2008. All procedures involving human subjects/patients were approved by University Institutional Ethics Committee (Approval # IEC-AIMS-2021-CW141). As the study includes an online questionnaire, participants were provided with the Informed Consent at the beginning of the questionnaire. Prior to the survey questions, participants were provided with consent selections to confirm their willingness to participate in the study, followed by a question to affirm their age as 18 or older.

### Participants

Convenience sampling was conducted through an online, digital survey link sent to the email database of a university in southern India. The link recipients included staff and students who were invited to share the link across social groups with encouragement to include university students. Inclusion criteria included students residing in India during the study period, aged 18 or older. Those who were not students, not living in India during the time of the study, and not aged 18 or older were excluded from this study. This resulted in 536 qualified participants: 246 (46%) students from high school, and 290 (36.9%) students in higher education studies (bachelor’s through doctoral degree studies 17.2%). Participants were aged between 18 and 49 years (*M* = 21.53, *SD* = 3.43) of which 61% (*n* = 327) were female and 39% were male (*n* = 209).

### Research design

This is a cross-sectional, exploratory, anonymous, web-based digital questionnaire study. The researchers aimed to identify factors that influenced suicide ideation among students during India’s 2nd Wave of the COVID-19 pandemic. To this end, a questionnaire was developed to capture specific variables.

### Measures

#### Independent variables

We created three categories of independent variables: 1) Sociodemographic, 2) COVID-19-specific variables, and 3) Clinical variables. The sociodemographic variables included age, gender, education, rural or urban residence, exercise, economic status, and marital status. The COVID-19 variables assessed online class challenges, concerns about viral contagion, fear of the 1st and 2nd waves of the pandemic, family challenges, vaccination status, and future expectations. The Clinical variables included psychological factors that can influence suicide ideation, including sad mood, fear, sleep disturbance, hopelessness, feeling like a failure, loss of interest, and loneliness.

The sociodemographic variables were ranked on a nominal scale (e.g., marital status) or ordinal scale (e.g., economic status). COVID-19 and Clinical variables were assessed using ordinal scales (Likert scales, e.g., 1 = not at all, 5 = very much). For the Binary Logistic Regression analysis, we recoded the suicide ideation variable, and relevant independent Clinical variables to binary variables.

#### Clinical variables selected

We included the following variables in our study because research has demonstrated their influence on suicide behaviors.

*Sleep disturbance*. Students with sleep disturbance have significantly higher levels of suicide ideation than those without [54–57]. Insomnia is independently and significantly associated with higher intensity of SI over and above other core depression symptoms. Sleep disturbance can also indicate the need for a further SI evaluation [54]. For these reasons we included sleep disturbance as a possible predictor of SI in our study.

*Sad mood and hopelessness*. Maladaptive sad moods can be long-standing and can influence suicidal thoughts [55,56]. Both high and low-income countries report adolescent sadness to be alarmingly pervasive. For example, two national surveys in Vietnam conducted in 2003 and 2009 (combined *N >* 10,000 adolescents) found an increase in adolescent sadness from 34% to 37.34% respectively, and in suicide behaviors from 5.28% to 12.21% respectively [57]. Likewise, independent studies conducted in south Korea and Malaysia found strong associations between sadness and suicidal behaviors [58,59]. In high-income countries, 31.5% to 35.8% of adolescents reported persistent sadness and hopelessness, and 18% seriously considered suicide in the prior 12 months [60,61]. Students with suicidal ideation also report higher levels of hopelessness [62,63], therefore we included these variables in our study.

*Loneliness*. Whereas social support can be a protective factor for suicide behaviors, loneliness is a determinant [64], contributing to suicidal thoughts and attempts [64–66]. Experiencing both depression and loneliness increases the risk for suicide ideation and attempts [66–73]. As students were separated from friends and classmates for protracted periods of time during the pandemic, loneliness is an included variable in our study as a possible contributor to SI.

*Economic stress*. Economic crises are associated with elevated suicide ideation, attempts, and deaths [74–76]. The pandemic stay-at-home orders led to suicides related to job loss and financial crisis exacerbated by fear of infection, loneliness, anxiety, and depression [77–80]. Of 153,000 suicides in India during 2020, one fourth occurred among daily wage workers who typically receive less pay [81]. Tragically, during the pandemic 272 family suicidal deaths occurred in India related to economic crisis and loss of a home [82]. For these reasons we included economic stress as a viable variable in our study. We now introduce our dependent variable for this study.

*Dependent variable*. Suicide ideation is the dependent variable in this study. We define it according to the 9-item Patient Health Questionnaire (PHQ-9): thoughts of self-harm; or thoughts that one would be better if dead. We further define this form of SI as ‘passive’ in line with Franklin [83], in that it implies a degree of attempt that is not explicitly communicated, as opposed to ‘active’ SI that implies an explicitly expressed intent. We recoded SI into a binary variable for the Logistic Binary Logistic Regression analysis.

#### Timing of study

The 2nd Wave began in March 2021, a period during which pandemic cases and mortalities exponentially surged. This wave began to subside by July 2021. The survey was completed within one month, during June 2021.

### Data analysis

Statistical analysis was conducted with SPSS, version 26. Descriptive statistics assisted in summarizing the characteristics of the data (means, standard deviations, percentages, and distribution of data visually through charts and graphs). Spearman Rank-Order Correlation Coefficient indicated the strength and direction of association between specific COVID-19 ordinal variables to help us understand their impact upon students during the 2nd wave. It also revealed the association between these COVID-19 variables and suicide ideation. The Chi-Square of Independence examined the strength of association between SI and categorical variables (age groups, gender). The Binary Logistic Regression was performed initially to identify individual variables that were significantly associated with the suicide ideation outcome variable (SI). Those individual variables that were significantly related to SI were then applied to a Binary Logistic Regression model to determine how much each of these contributed to, or predicted, the presence of suicide ideation among the student sample.

## Results

### Objective 1

What are the primary socio-demographic, covid-related, and Clinical predictors of SI in our sample of students?

Five-hundred thirty-six students participated in this study. The mean age was 21.53 (*N* = 536; *SD* = 3.43, range = 18–49) of which 61% were female and 39% male. Suicide ideation point prevalence was 48.7%. Prevalence of SI among the females was 49.8% (*n* = 327), and within the sample of males it was 46.8% (*n* = 209). SI was higher among those completing 12^th^ grade (52.4%) than those with university education (45.5%). Most respondents were single (91.8%), living in their family home (81.7%), and most self-identified as financially poor (55.6%). Table 1 provides frequencies of socio-demographic and COVID-19 specific variables.

**Table 1:** Frequencies of socio-demographic and COVID-19 concerns.

Sociodemographic Variable	%	N	Sociodemographic Variable	%	N
**Gender**			**Concern - 1st wave COVID**		
Male	39	209	Not at all	3	16
Female	61	327	Slightly	8	43
**Highest Edu Completed**			Somewhat	23.1	124
Primary School	0	0	Very	27.4	147
10th standard (grade)	0.4	2	Extremely	38.4	206
! 2th standard (grade)	45.5	244	**Total—some concern**	97	
Bachelor’s degree	36.9	198	**Total—very to extreme**	**65.8**	
Master’s degree	15.1	81	**Concern - 2nd wave**		
Doctoral degree	2.1	11	Not at all	1.1	6
**Marital Status**			Slightly	2.8	15
Married/Living together	3.4	18	Somewhat	13.8	74
Divorced/Separated	0.2	1	Very	24.1	129
Relationship living apart	4.7	25	Extremely	58.2	312
Single	91.8	492	**Total—some concern**	98.9	
**Rural or Urban**			**Total-very to extr concern**	**82.3**	
Rural	27.4	147	**Concern—contracting virus**		
Semi-urban	32.1	172	Not at all	8.8	47
Urban	39.7	213	Slightly	13.6	73
Other	0.7	4	Somewhat	23.9	128
**Type of Dwelling**			Very	29.9	160
Family home	81.7	438	Extremely	23.9	128
Renting	10.6	57	**Total—some concern**	**91.2**	
Student housing	6.7	36	**Total–No concern**	8.8	
Other	0.9	5	**Vaccine status**		
**Exercise since pandemic**			Have had no vaccine	85.6	459
Stopped completely	22.8	122	Had first vaccine	10.1	54
Decreased	28.9	155	Have had two vaccines	4.3	23
Remained the same	10.8	58	**Expectations about future**		
Increased	16.6	89	Very hopeless	8.8	47
None before or now	20.9	112	Slightly hopeless	20.1	108
**Economic State COVID**			Uncertain	35.8	192
**POOR**			Hopeful	19.6	105
Extremely poor	7.6	41	Very hopeful	15.7	84
Somewhat poor	23.5	126	**More fear since pandemic**		
Poor	24.4	131	Not at all	9	48
**Total Poor**	**55.6**	**298**	Slightly	12.1	65
**GOOD**			Moderate	26.7	143
Good financial state	11.9	155	Very	31.2	167
Very good	28.9	64	Extremely	21.1	113
Extr good financially	3.5	19	**Total—mod to extreme**	79	
**Total Good Financially**	**44.4**	**238**	**Total—severe to extreme**	**52.3**	

Seven percent of the students lost a loved one to COVID-19, and 69.2% knew of someone who had COVID-19. More than 90% were concerned about contracting COVID-19.

Regarding age and gender, suicide ideation among those aged 21 and below, and those 22 and above was 51.9% and 44.1% respectively. Chi-square for independence tests (with Yates’ Continuity Correction) indicated no significant association between suicide ideation and these two age groups: *X*^2^(1, *n* = 536) = .29, *p* = .09, *phi* = .08, and no significant association between SI and gender: *X*^2^(1, *n* = 536) = .34, *p* = .56, *phi* = -.03.

**Fig 2:**
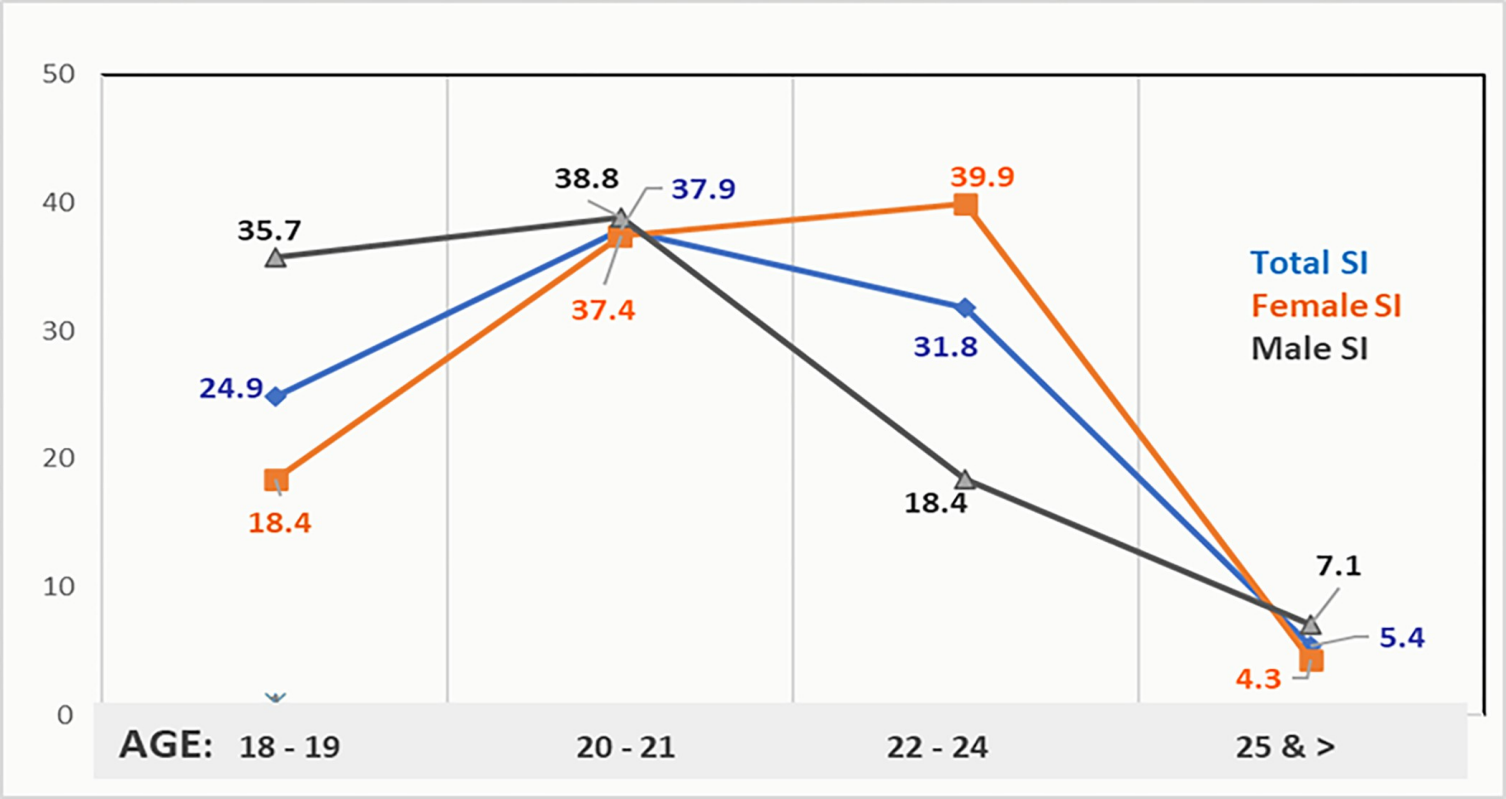
Suicide ideation rates by age and gender.

To better understand the relationships between age, gender, and SI we created further divisions (Fig 2): a) aged 18 to 19, b) 20 to 21, c) 22 to 24, and d) 25 and above. The outcomes were striking with SI spiking and converging across gender among those aged 20 to 21, then dropping dramatically to 5.4% among those aged 25 and older. Females aged 22 to 24 had the highest rate of SI (39.9%).

**Fig 3:**
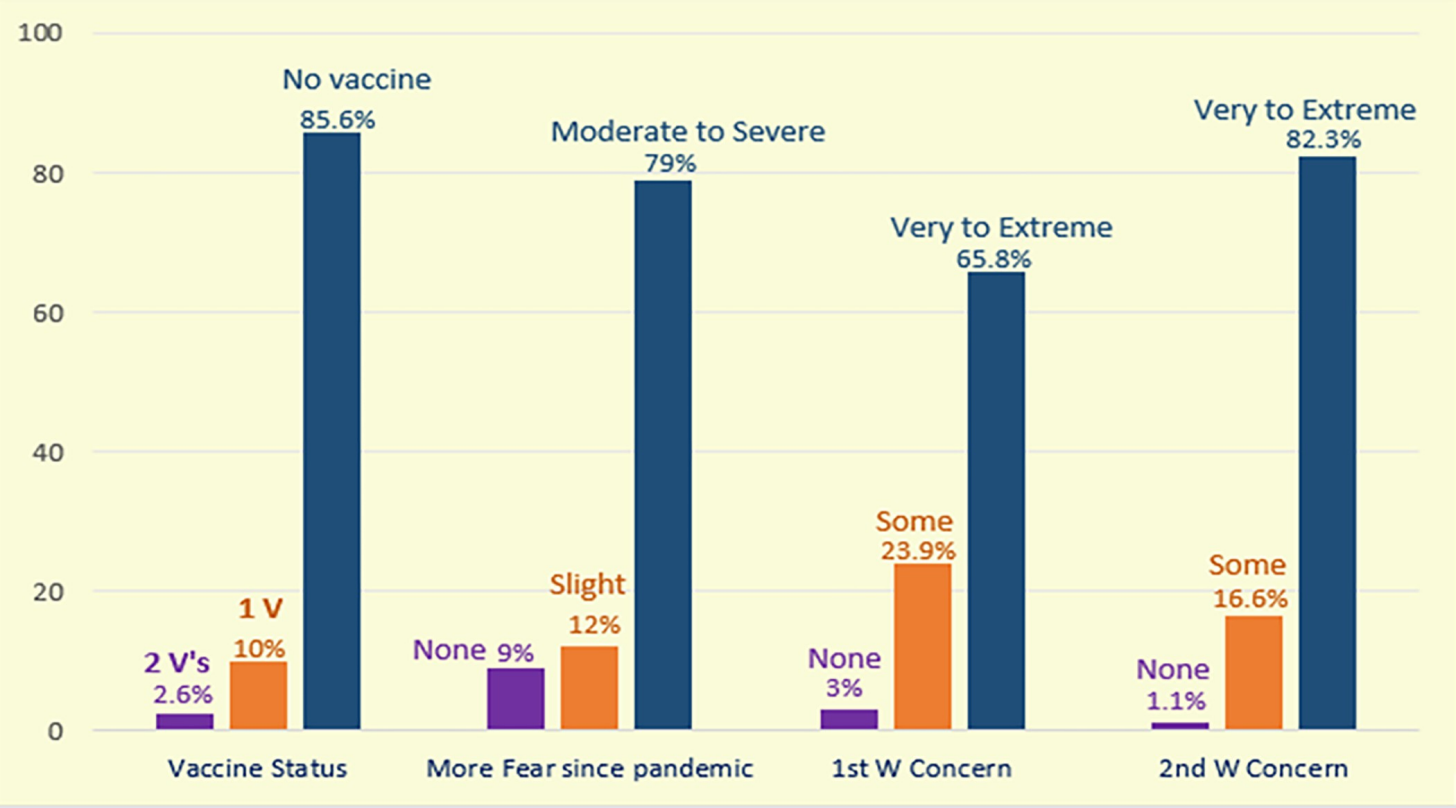
Pandemic-related student vaccines, fear, and concerns.

Alarmingly, as Fig 3 shows below, 85.6% of respondents had not received any vaccine more than one year into the pandemic. Of these, 49% reported suicide ideation. Nearly 80% of the participants reported moderate to severe fear related directly to the pandemic (Fig 3). Further, most experienced “very-to-extreme” concern during India’s 1st wave and 2nd wave of COVID-19.

#### Correlates of suicide ideation

We used the Spearman Rank Order correlation to assess the relationship between COVID-19 risk factors and suicide ideation as is shown in Table 2 (*n* = 536). As hypothesized, “suicide Ideation” was positively and moderately associated with “More fear since COVID” (*r* = .29, p < .0001) and with “Loneliness” (*r* = .20, p < .0001) but was not significantly related to the other variables in the table.

**Table 2:** Spearman rank-order correlations among COVID-19 related variables.

		2	3	4	5	6	7	8
**Suicide Ideation**	1	.293^***^	-.066	.016	.013	.200^***^	-.001	-.013
**Lonely since covid**	2	1	-.106^*^	-.034	.055	.221^***^	.088^*^	.129^**^
**Future expectations**	3		1	.296^***^	-.059	-.113^**^	.058	-.096^*^
**Economic security**	4			1	-.062	-.105^*^	.008	-.099^*^
**Contract covid fear**	5				1	.500^***^	**.28** ^***^	**.294** ^***^
**More fear since covid**	6					1	.176^***^	**.338** ^***^
**1st wave worry**	7						1	**.398** ^***^
**2nd wave worry**	8							1

Correlation is significant at the .05 level *; at the .001 level: **, at the .0001 level ***.

We then examined relationships between several pandemic-related variables using Spearman’s rank-order correlation. A strong association emerged between “More fear since COVID” and a “Fear of contracting the virus.” Similarly, there were moderately strong associations between “2nd Wave Worry” and the following variables: “More fear since the pandemic,” “Fear of contracting the virus,” and “Fear of the 1st Wave” all of which suggest that fear and worry were consistently strong in the minds of the participants during the 2nd Wave. These associations are in bold in the table.

As we might anticipate, “Future expectations” was significantly and positively associated with “Economic security” suggesting that those who had reliable financial resources were more likely to feel positive about the future. On the other hand, “Future expectations” was negatively and significantly associated with “More fear since the pandemic,” and “More fear of the 2nd Wave” (both with small effect sizes) which could suggest that those with unreliable income experienced more fear as the virus persisted.

#### Individual binary logistic regression

Variables were recoded into binary variables for the Individual Binary Logistic Regression. This test was helpful in examining the predictive power of individual COVID-related variables (e.g., fear of covid, vaccine status, online classes) and individual Clinical values (sad mood, sleep disturbance, and others) on suicidal thoughts. Results indicated that most sociodemographic and pandemic-specific variables were not predictors of SI, including online classes, nor were the effect sizes strong. For example, of those participants (70%) who reported no family challenges, 46.9% experienced suicide ideation, whereas those with family challenges also reported SI (52.5%). As shown in Table 3 below, the difference between the two groups was not significant and the effect size was very small (*r* = .05, *p* = .278). Reducing or stopping exercise routine increased the odds of experiencing SI (Odds Ratio/OR = 1.59), but the effect size was small (*r* = .110, *p* < .05). The variable of “more fear” since COVID-19 was not statistically associated with SI (CI = 98–3.38, *p* = .07) in the individual odds ratio (OR). Hence, variables that were either unrelated to SI, or that had only weak associations to SI were not included in our final model using Binary Logistic Regression.

**Table 3:** Suicide ideation odds ratios pertaining to individual variables.

Variable	N	Ratio	Suicidal Ideation		OR-Odds Ratio	95% CI	P value	Effect Size
			N	Per. %				
**Gender**								
0 = Male	209	.39	98	46.8	.06	.887–1.7	.562	.029
1 = Female	327	.61	163	49.8				
**Age**								
0 = Up to 21	316	59	164	51.9	1.01	.71–1.41	1.00	.001
1 = 22 and above	220	41	97	44.1				
**Highest Educ**								
0 = Up to 12th	246	46.0	129	52.4	.75	.54–1.06	.120	.071
1 = UG—PG	290	54.0	132	45.5				
**Rural or Urban**								
0 = Rural	147	27.5	78	53.1	.782	.53–1.14	.240	-.055
1 = Urban/other	389	72.5	183	46.9				
**Type of Dwelling**								
0 = Other	98	18.3	47	48	1.03	.67–1.60	.977	.006
1 = Family home	438	81.7	214	48.7				
**Exercise Routine**								
0 = same or more	146	34.5	60	41.1	1.597	1.07–2.4	.030	.110
1 = less or none	277	65.4	146	52.7				
**Economics: Covid**								
0 = No problems	237	44.3%	117	49.4	.946	.67–1.33	.818	-.014
1 = Finance prob	299	55.7	144	48.0				
**1st wave concern**								
0 = No worry	59	11.0%	23	39	1.55	.89–2.67	.153	.068
1 = Yes worried	477	89.0%	238	49.8				
**2nd wave concern**								
0 = No worry	21	4.0	13	61.9	.57	.23–1.40	.307	-.054
1 = Yes worried	515	96.0	247	48.1				
**Concern—COVID**								
0 = No worry	47	8.8	22	46.8	1.086	.59–1.98	.90	.011
1 = Yes worried	489	91.2	239	48.9				
**Vaccine status**								
0 = Yes Vaccine	77	14.4	36	46.8	1.095	.67–1.77	.81	.06
1 = No Vaccine	459	85.6	225	49				
**Fut. expectations**								
0 = Hopeful	189	35.1	87	46.3	1.154	.81–1.65	.484	.034
1 = Hopeless	347	64.9	173	49.9				
**Online Classes**								
0 = No	215	40.1	113	52.6	.772	.546–1.1	.17	.063
1 = Yes	321	59.9	148	56.7				
**Family Challenges**								
0 = No	375	70.1	176	46.9	1.250	.86–1.81	.278	.051
1 = Yes	161	29.9	84	52.5				
**Fear of death**								
0 = No	451	84.3	217	48.1	.13	.71–1.80	.69	.02
1 = Yes	85	15.7	43	51.2				
**Hopelessness**								
0 = not hopeless	189	35.3%	88	46.6	1.14	.80–1.63	.52	.03
1 = hopeless	347	64.7	173	49.9				
**Loneliness**								
0 = No	54	10.1	10	18.5	4.86	2.4–9.72	.000	.20
1 = Yes	482	89.9	251	52.1				
**Sleep disturbance**								
0 = No	118	22.1	20	16.9	6.67	3.97–11.	.0001	.337
1 = Yes	418	77.9	240	57.6				
**Sad mood**								
**0—no**	141	26.4	20	14.2	9.47	5.7–15.8	.0001	.413
**1—yes**	395	73.7	241	61				
**Loss of interest**								
0 = No	119	22.2	18	15.1	7.84	4.5–13.4	.000	.36
1 = Yes	417	77.8	243	58.3				
**Feel like Failure**								
**0 =** No	171	**32**	28	16.4	9.02	5.7–14.3	.0001	.443
1 = Yes	365	68	233	63.87				
**More fear: COVID**								
0 = No	113	21.1	39	34.5	1.82	.98–3.38	.07	.146
1 = Yes	423	78.9	221	52.4				

#### Logistic regression model

The strongest individual ORs relative to SI were sad mood, feeling like a failure, loneliness, sleep disturbance, loss of interest (apathy) as indicated at the bottom of Table 3. We applied these five independent variables in a Binary Logistic Regression. The full model was statistically significant, *X*^2^ (5, N = 536) = 183.1, *p* < .0001, indicating that it was able to distinguish between respondents who reported and did not report suicide ideation. The model explained between 28.9% (Cox & Snell R^**2**^) and 38.6% (Nagelkerke R^**2**^) of the variance of suicide ideation, and correctly classified 75.7% of cases. As shown in Table 4, all five of the independent variables made a unique statistically significant contribution to the model. The strongest predictor was fear of failure, which increased the odds of experiencing suicidal thoughts 4.2-fold. The other model variables were also strong predictors of SI as follows:

- Sleep disturbance: 3-fold increase in the odds having SI
- Persistent sad mood: 2.9-fold increase in the odds having SI
- Loneliness: 2.8-fold increase in the odds having SI
- Loss of interest: 2.6 times the odds having SI

**Table 4:** Variables in the equation—multiple variables.

	B	S.E.	Wald	df	Sig.	OR	95% C.I.	
							Lower	Upper
**Loss of Interest**	.955	.327	8.551	1	.003	2.600	1.370	4.932
**Sad, low mood**	1.064	.310	11.780	1	.001	2.899	1.579	5.324
**Feeling of failure**	1.428	.260	30.197	1	.000	4.169	2.506	6.938
**Lonely**	1.017	.422	5.820	1	.016	2.765	1.210	6.318
**Sleep disturbance**	1.112	.305	13.320	1	.000	3.040	1.673	5.523
**Constant**	-4.561	.572	63.488	1	.000	.010		

### Objective 2

Do these predictors help to support the first step of the 3-Step Theory of suicide ideation in terms of combined hopelessness and pain?

Recall that the 3ST asserts that suicide ideation is likely to occur if one experiences both pain and hopelessness. In terms of pain, students reported pandemic-related sad mood, loneliness, fear of the pandemic and of contracting the virus, feeling like a failure, sleep disturbance, and fear and worry, all of which fall under 3ST’s pain category. Additionally, sad mood (psychological pain) was significantly associated with suicide ideation in the individual OR (Table 3: *r* = .413, p < .0001). However, hopelessness, reported by 64.7% of the respondents, was not significantly associated with SI (Table 3).

We explored this more deeply and found that out of 536 participants, 35.3% (*N* = 189) had no sense of hopelessness, but many of these reported high levels of pandemic-related fear. Of those with no hopelessness, 16.4% (*N* = 88) experienced suicide ideation, and their reported fear was very high with 94.3% expressing concern about India’s 1st Wave of the pandemic, and 95.5% reporting concern (somewhat to extreme) about India’s 2nd Wave. Further, 94.3% feared contracting COVID-19, and 95.5% felt more fear since the arrival of the pandemic. Overall, a modest subset of students who experienced psychological pain but who did not experience hopelessness reported SI. Fear appeared to be the dominant factor affecting them. Hence, Step 1 of the 3ST was not fully supported by our study, although most of the participants with SI also felt hopelessness.

## Discussion

Suicide ideation levels were inordinately high, 48.7%, among students within India during the pandemic’s 2nd wave. Participants struggled with COVID-19-related variables that convey worry and fear, including online classes, low vaccine status, economic vulnerability, and fear of viral contagion. Our supposition that the variables: “fear of the virus” and “economic hardship” would predict suicide ideation was not statistically supported. This is likely because even those who were economically secure reported suicidal thoughts. In our study the odds of having suicide ideation were predicted by sleep disturbance, sad mood, loneliness, apathy, and feelings of failure. The findings are important given the high level of SI reported by students.

Why was SI so high among students in this study? There are several possibilities. First, 68% of the participants experienced a sense of failure, the strongest predictor of suicide ideation in this study. During the pandemic, school closure, perceived stress, and fear of viral infection all contributed to scholastic stress [84]. Studies reported that students faced online class obstacles. These included unreliable electricity, accessibility, networks, and lack of digital skills. This led to social isolation, boredom, frustration, poorer academic performance, and lethargy [85]. This confluence of frustrations could understandably lead to a perceived sense of failure.

Second, positive, supportive peer and family relationships among youth are associated with declines in SI [86], but the pandemic lockdowns prohibited in-person gatherings. Loneliness was reported by nearly all students (90%) in this study across gender, suggesting inadequate or absent supportive connections. Loneliness, a public health concern, can predict suicidal thoughts, mental and physical illnesses, and death [87,88]. We had predicted that loneliness would be among the top predictors of SI, and it was, indeed, a strong predictor, but not the strongest. Still, loneliness was strongly associated with SI.

Finally, a completely unprecedented change in lifestyle was, by necessity, imposed upon the public by the unparalleled convergence of atypical negative life events: enforced quarantines, lockdowns, financial stress, school closures, loss of work, social isolation, and fear of a silent, potentially fatal virus quietly racing around the world. These events, along with witnessing millions becoming ill and dying, potentially intensified to a crescendo of exceptional levels of stress, worry, fear, and suicide ideation among the students. Humanity was not prepared for such a catastrophic world event. Yet we have no excuse not to be prepared for the next such event.

### Implications for research and practice

Ultimately, we must take a step back and reevaluate the current mental health of young people worldwide considering the high prevalence of SI among youth during the Second Wave: Why are thoughts of suicide plaguing so many young people? What coping mechanisms are they using to deal with tense circumstances, negative thoughts, and challenging feelings? Do they have someone they can confide their pleasures and sorrows in who is positive and safe? What is the nature of their relationships with peers, teachers, and family members?

The rise in suicide ideation suggests that today’s youth have not internalized strong, healthy coping mechanisms for dealing with life’s inevitable challenges. When constructive techniques are lacking, self-criticism can lead to suicidal ideation and other poor decisions such self-harm, excessive internet use, or substance use. Our findings suggest the importance of developing policy-supported robust, positive interventions that can strengthen our youth psychologically and reduce suicidal thoughts and behaviors. Interventions should increase their resiliency when facing unpredictable life events, including future disasters. Positive, supportive parental involvement buffers youth from suicidal thoughts, hence parents should be included in such initiatives. Additionally, community mental health centers, schools, medical offices, and hospital emergency departments should train personnel in standardized suicide behaviors assessments [89] along with overall mental health assessments.

### Uniqueness of this study

This study is unique as it captures an unprecedented global historical period during which socioeconomic, behavioral, and other restrictions were government mandated. The irony of these intended safeguards is that they precipitated varying degrees of stress depending upon one’s context. The authors are not aware of another historical period during which prevalence rates of suicide ideation were so high among a general student population. In 2008 a study in India reported a “high” student SI rate of 15.8%, which *was* high at that time [90]. Now 16 years later, it is exceptionally high at 48.7%.

What is clear about studies examining suicide ideation and potential predictors is that this relationship is often multifaceted and complex. At times SI is contextually influenced (e.g., pandemic, economic recessions, family adversities), and at times more psychologically initiated (e.g., feeling like a failure). We see these two categories as mutually interdependent.

### Limitations

This study did not include variables such as a personal or family history of suicidal attempts/deaths, or excessive online use, all of which can influence SI. Given the unique scenario of India’s 2nd wave, the cross-sectional design, the online, non-randomized convenience sampling, and self-report data (which researchers debate regarding reliability), results are not generalizable to a larger and/or different population. Overall, there are certainly important lessons to be learned from the findings in this study conducted during an unprecedented, threatening, world pandemic.

## Abbreviations

SI: Suicide Ideation
3ST: Three-Step Theory of suicide behaviors
NCRB: National Crime Record Bureau
OR: Odds Ratios
